# (*E*)-6-Chloro-*N*′-(3,5-dichloro-2-hydroxy­benzyl­idene)­nicotinohydrazide

**DOI:** 10.1107/S1600536809007272

**Published:** 2009-03-06

**Authors:** Chun-yan Ren

**Affiliations:** a College of Chemistry and Pharmacy, Qingdao Agricultural University, Shandong 266109, People’s Republic of China

## Abstract

The title Schiff base compound, C_13_H_8_Cl_3_N_3_O_2_, was synthesized by the condensation reaction of 3,5-dichloro­salicyl­aldehyde with 6-chloro­nicotinic acid hydrazide in 95% ethanol. The mol­ecule is nearly planar, with a dihedral angle of 1.9 (2)° between the aromatic ring planes, and an intra­molecular O—H⋯N hydrogen bond is observed. In the crystal, the mol­ecules are connected by inter­molecular N—H⋯O hydrogen bonds into infinite chains propagating in [100].

## Related literature

For general background, see: Kim *et al.* (2005 ▶); Fan *et al.* (2007 ▶). For background on the biological activities of Schiff bases, see: Ren *et al.* (2002 ▶); Takeuchi *et al.* (1998 ▶). For a related structure, see: Zhi (2008 ▶). For reference structural data, see: Allen *et al.* (1987 ▶).
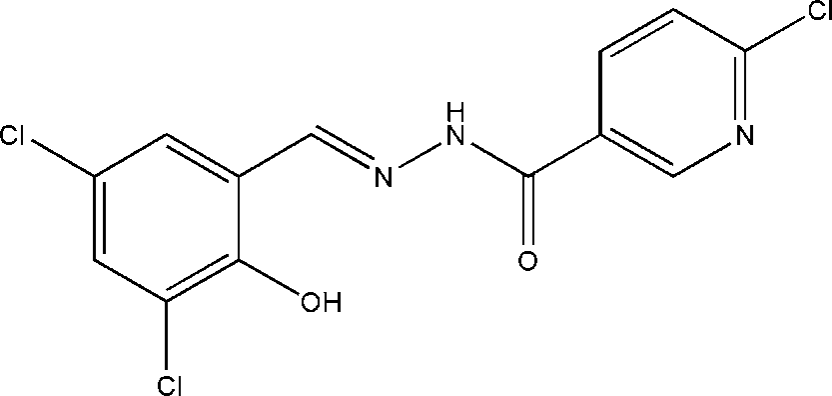


## Experimental

### 

#### Crystal data

C_13_H_8_Cl_3_N_3_O_2_
*M* *_r_* = 344.57
Monoclinic, 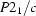

*a* = 4.8920 (10) Å
*b* = 18.014 (4) Å
*c* = 16.112 (3) Å
β = 97.90 (3)°
*V* = 1406.4 (5) Å^3^
*Z* = 4
Mo *K*α radiation
μ = 0.66 mm^−1^
*T* = 298 K
0.27 × 0.23 × 0.23 mm

#### Data collection

Siemens SMART CCD diffractometer
Absorption correction: multi-scan (*SADABS*; Siemens, 1996 ▶) *T*_min_ = 0.842, *T*_max_ = 0.864
7275 measured reflections
2478 independent reflections
1323 reflections with *I* > 2σ(*I*)
*R*_int_ = 0.078

#### Refinement

*R*[*F* ^2^ > 2σ(*F* ^2^)] = 0.049
*wR*(*F* ^2^) = 0.114
*S* = 1.01
2478 reflections
191 parameters
H-atom parameters constrained
Δρ_max_ = 0.25 e Å^−3^
Δρ_min_ = −0.40 e Å^−3^

### 

Data collection: *SMART* (Siemens, 1996 ▶); cell refinement: *SAINT* (Siemens, 1996 ▶); data reduction: *SAINT*; program(s) used to solve structure: *SHELXS97* (Sheldrick, 2008 ▶); program(s) used to refine structure: *SHELXL97* (Sheldrick, 2008 ▶); molecular graphics: *SHELXTL* (Sheldrick, 2008 ▶); software used to prepare material for publication: *SHELXTL*.

## Supplementary Material

Crystal structure: contains datablocks global, I. DOI: 10.1107/S1600536809007272/hb2920sup1.cif

Structure factors: contains datablocks I. DOI: 10.1107/S1600536809007272/hb2920Isup2.hkl

Additional supplementary materials:  crystallographic information; 3D view; checkCIF report

## Figures and Tables

**Table 1:** Hydrogen-bond geometry (Å, °)

*D*—H⋯*A*	*D*—H	H⋯*A*	*D*⋯*A*	*D*—H⋯*A*
N2—H2⋯O2^i^	0.86	2.20	2.930 (4)	142
O1—H1⋯N1	0.82	1.82	2.540 (4)	147

Symmetry code: (i) 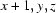.

**Fig. 1.**
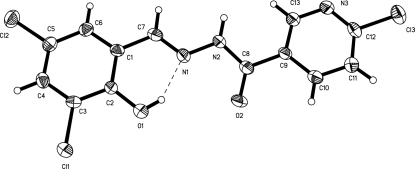
The molecular structure of (I), with displacement ellipsoids drawn at the 30% probability level. The dashed lines indicate hydrogen bonds.

**Fig. 2.**
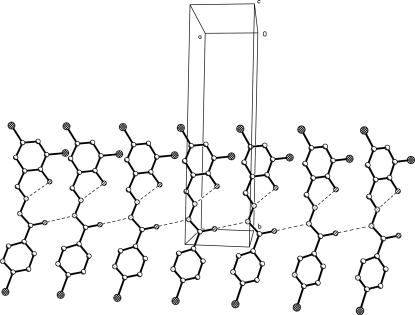
The infinite chains structure formed via hydrogen bonds, H atoms have been omitted for clarity. The dashed lines indicate the connections between the donor and acceptor atoms of the hydrogen bonds.

## supplementary crystallographic information

### Comment

Schiff base compounds have been widely investigated over a century (Fan *et al.*, 2007; Kim *et al.*, 2005). Some of the complexes derived from Schiff bases have been found to have pharmacological and antitumor properties (Ren *et al.*, 2002; Takeuchi,*et al.*, 1998). In this paper, the crystal structure of the title compound, (I), a new Schiff base compound derived from the condensation reaction of 3,5-dichlorosalicylaldehyde with 6-chloronicotinic acid hydrazide is reported.

The molecule of (I) displays a *trans* configuration with respect to the C=N and C—N bonds (Fig. 1). All the bond lengths are within normal ranges (Allen *et al.*, 1987), and are comparable to those in the related compound 6-chloro-*N'*-(2-hydroxy-1-naphthylmethylene)nicotinohydrazide (Zhi 2008). The Schiff base molecule is nearly planar, with a dihedral angle between the benzene ring and the pyridine ring of 1.9 (2)°. An intramolecular O—H···N hydrogen bond is observed. The molecules are connected *via* intermolecular N—H···O hydrogen bonds into infinite chains along the *a* axis (Table 1, Fig. 2).

### Experimental

3,5-Dichlorosalicylaldehyde (0.1 mmol, 19.0 mg) and 6-chloronicotinic acid hydrazide (0.1 mmol, 17.1 mg) were dissolved in a 95% ethanol solution (10 ml). The mixture was stirred at room temperature to give a clear colorless solution. Light yellow blocks of (I) were formed by gradual evaporation of the solvent over a period of five days at room temperature.

### Refinement

All H atoms were placed in geometrically idealized positions, with C—H = 0.93 Å, O—H = 0.82 Å and N—H = 0.86 Å and refined as riding with *U*_iso_(H) = 1.2*U*_eq_(C,*N*) or 1.5*U*_eq_(O).

### Crystal data

**Table:**

C_13_H_8_Cl_3_N_3_O_2_	*F*(000) = 696
*M**_r_* = 344.57	*D*_x_ = 1.627 Mg m^−^^3^
Monoclinic, *P*2_1_/*c*	Mo *K*α radiation, λ = 0.71073 Å
Hall symbol: -P 2ybc	Cell parameters from 669 reflections
*a* = 4.892 (1) Å	θ = 2.6–18.8°
*b* = 18.014 (4) Å	µ = 0.66 mm^−^^1^
*c* = 16.112 (3) Å	*T* = 298 K
β = 97.90 (3)°	Block, light yellow
*V* = 1406.4 (5) Å^3^	0.27 × 0.23 × 0.23 mm
*Z* = 4	

### Data collection

**Table:**

Siemens SMART CCD diffractometer	2478 independent reflections
Radiation source: fine-focus sealed tube	1323 reflections with *I* > 2σ(*I*)
graphite	*R*_int_ = 0.078
φ and ω scans	θ_max_ = 25.1°, θ_min_ = 1.7°
Absorption correction: multi-scan (*SADABS*; Siemens, 1996)	*h* = −5→5
*T*_min_ = 0.842, *T*_max_ = 0.864	*k* = −15→21
7275 measured reflections	*l* = −19→15

### Refinement

**Table:**

Refinement on *F*^2^	Primary atom site location: structure-invariant direct methods
Least-squares matrix: full	Secondary atom site location: difference Fourier map
*R*[*F*^2^ > 2σ(*F*^2^)] = 0.049	Hydrogen site location: inferred from neighbouring sites
*wR*(*F*^2^) = 0.114	H-atom parameters constrained
*S* = 1.01	*w* = 1/[σ^2^(*F*_o_^2^) + (0.0343*P*)^2^ + 0.2069*P*] where *P* = (*F*_o_^2^ + 2*F*_c_^2^)/3
2478 reflections	(Δ/σ)_max_ = 0.001
191 parameters	Δρ_max_ = 0.25 e Å^−^^3^
0 restraints	Δρ_min_ = −0.40 e Å^−^^3^

### Special details

**Table:**

Geometry. All e.s.d.'s (except the e.s.d. in the dihedral angle between two l.s. planes) are estimated using the full covariance matrix. The cell e.s.d.'s are taken into account individually in the estimation of e.s.d.'s in distances, angles and torsion angles; correlations between e.s.d.'s in cell parameters are only used when they are defined by crystal symmetry. An approximate (isotropic) treatment of cell e.s.d.'s is used for estimating e.s.d.'s involving l.s. planes.
Refinement. Refinement of *F*^2^ against ALL reflections. The weighted *R*-factor *wR* and goodness of fit *S* are based on *F*^2^, conventional *R*-factors *R* are based on *F*, with *F* set to zero for negative *F*^2^. The threshold expression of *F*^2^ > σ(*F*^2^) is used only for calculating *R*-factors(gt) *etc*. and is not relevant to the choice of reflections for refinement. *R*-factors based on *F*^2^ are statistically about twice as large as those based on *F*, and *R*- factors based on ALL data will be even larger.

### Fractional atomic coordinates and isotropic or equivalent isotropic displacement parameters (Å^2^)

**Table:**

	*x*	*y*	*z*	*U*_iso_*/*U*_eq_	
Cl1	0.3842 (2)	0.62724 (6)	0.45177 (7)	0.0534 (3)	
Cl2	1.2166 (3)	0.50424 (7)	0.65897 (7)	0.0651 (4)	
Cl3	1.2331 (3)	1.28010 (7)	0.69130 (8)	0.0698 (4)	
N1	0.9665 (7)	0.8466 (2)	0.6071 (2)	0.0471 (9)	
N2	1.0608 (7)	0.91708 (18)	0.6266 (2)	0.0474 (9)	
H2	1.2318	0.9261	0.6437	0.057*	
O1	0.6119 (6)	0.76626 (15)	0.51866 (18)	0.0542 (8)	
H1	0.6800	0.8046	0.5397	0.081*	
O2	0.6214 (6)	0.95721 (16)	0.59872 (19)	0.0619 (9)	
C1	1.0059 (8)	0.7165 (2)	0.6081 (2)	0.0408 (10)	
C2	0.7633 (8)	0.7076 (2)	0.5502 (2)	0.0399 (10)	
C3	0.6750 (8)	0.6374 (2)	0.5262 (2)	0.0411 (11)	
C4	0.8115 (8)	0.5751 (2)	0.5595 (3)	0.0455 (11)	
H4	0.7479	0.5280	0.5428	0.055*	
C5	1.0456 (9)	0.5834 (2)	0.6182 (3)	0.0461 (11)	
C6	1.1444 (9)	0.6532 (2)	0.6417 (3)	0.0450 (11)	
H6	1.3036	0.6581	0.6801	0.054*	
C7	1.1106 (9)	0.7906 (3)	0.6314 (2)	0.0452 (11)	
H7	1.2817	0.7964	0.6639	0.054*	
C8	0.8673 (9)	0.9716 (2)	0.6171 (3)	0.0450 (11)	
C9	0.9672 (8)	1.0483 (2)	0.6324 (3)	0.0410 (10)	
C10	0.8110 (9)	1.1062 (2)	0.5936 (3)	0.0506 (12)	
H10	0.6519	1.0962	0.5566	0.061*	
C11	0.8935 (9)	1.1786 (3)	0.6102 (3)	0.0543 (12)	
H11	0.7949	1.2183	0.5842	0.065*	
C12	1.1270 (9)	1.1895 (2)	0.6666 (3)	0.0477 (12)	
N3	1.2807 (7)	1.1365 (2)	0.7059 (2)	0.0502 (10)	
C13	1.1988 (9)	1.0670 (2)	0.6874 (3)	0.0493 (11)	
H13	1.3051	1.0285	0.7133	0.059*	

### Atomic displacement parameters (Å^2^)

**Table:**

	*U*^11^	*U*^22^	*U*^33^	*U*^12^	*U*^13^	*U*^23^
Cl1	0.0452 (7)	0.0496 (7)	0.0632 (8)	−0.0075 (6)	0.0002 (5)	−0.0027 (6)
Cl2	0.0715 (9)	0.0529 (8)	0.0676 (8)	0.0141 (7)	−0.0015 (6)	0.0027 (6)
Cl3	0.0842 (10)	0.0520 (8)	0.0722 (9)	−0.0184 (7)	0.0071 (7)	−0.0060 (7)
N1	0.040 (2)	0.040 (2)	0.062 (3)	−0.0058 (19)	0.0091 (18)	−0.0038 (19)
N2	0.036 (2)	0.037 (2)	0.067 (3)	−0.0058 (19)	−0.0010 (18)	−0.0074 (19)
O1	0.0454 (19)	0.045 (2)	0.069 (2)	−0.0041 (16)	−0.0032 (15)	−0.0010 (16)
O2	0.0317 (19)	0.060 (2)	0.092 (2)	−0.0082 (16)	0.0027 (16)	−0.0115 (18)
C1	0.030 (2)	0.045 (3)	0.048 (3)	−0.004 (2)	0.010 (2)	−0.005 (2)
C2	0.034 (3)	0.035 (3)	0.050 (3)	−0.002 (2)	0.005 (2)	−0.001 (2)
C3	0.030 (2)	0.049 (3)	0.043 (3)	−0.002 (2)	0.0032 (19)	0.000 (2)
C4	0.048 (3)	0.038 (3)	0.053 (3)	−0.005 (2)	0.016 (2)	−0.003 (2)
C5	0.048 (3)	0.047 (3)	0.044 (3)	0.006 (2)	0.010 (2)	0.002 (2)
C6	0.041 (3)	0.049 (3)	0.045 (3)	0.005 (2)	0.007 (2)	−0.004 (2)
C7	0.035 (3)	0.053 (3)	0.048 (3)	−0.007 (2)	0.006 (2)	−0.008 (2)
C8	0.040 (3)	0.046 (3)	0.049 (3)	−0.008 (2)	0.006 (2)	−0.005 (2)
C9	0.033 (3)	0.041 (3)	0.049 (3)	−0.005 (2)	0.006 (2)	−0.005 (2)
C10	0.041 (3)	0.053 (3)	0.055 (3)	−0.008 (2)	−0.001 (2)	−0.001 (2)
C11	0.053 (3)	0.048 (3)	0.061 (3)	−0.003 (2)	0.003 (2)	0.006 (2)
C12	0.052 (3)	0.046 (3)	0.047 (3)	−0.015 (2)	0.013 (2)	−0.001 (2)
N3	0.043 (2)	0.048 (3)	0.057 (2)	−0.004 (2)	−0.0001 (18)	−0.006 (2)
C13	0.043 (3)	0.044 (3)	0.061 (3)	−0.002 (2)	0.004 (2)	−0.006 (2)

### Geometric parameters (Å, °)

**Table:**

Cl1—C3	1.739 (4)	C4—C5	1.389 (5)
Cl2—C5	1.736 (4)	C4—H4	0.9300
Cl3—C12	1.742 (4)	C5—C6	1.382 (5)
N1—C7	1.263 (5)	C6—H6	0.9300
N1—N2	1.372 (4)	C7—H7	0.9300
N2—C8	1.358 (5)	C8—C9	1.474 (5)
N2—H2	0.8600	C9—C13	1.380 (5)
O1—C2	1.349 (4)	C9—C10	1.391 (5)
O1—H1	0.8200	C10—C11	1.380 (6)
O2—C8	1.227 (5)	C10—H10	0.9300
C1—C6	1.396 (5)	C11—C12	1.373 (6)
C1—C2	1.414 (5)	C11—H11	0.9300
C1—C7	1.460 (5)	C12—N3	1.321 (5)
C2—C3	1.375 (5)	N3—C13	1.337 (5)
C3—C4	1.376 (5)	C13—H13	0.9300
			
C7—N1—N2	120.9 (4)	N1—C7—C1	119.3 (4)
C8—N2—N1	115.9 (4)	N1—C7—H7	120.3
C8—N2—H2	122.1	C1—C7—H7	120.3
N1—N2—H2	122.1	O2—C8—N2	121.3 (4)
C2—O1—H1	109.5	O2—C8—C9	122.0 (4)
C6—C1—C2	118.8 (4)	N2—C8—C9	116.7 (4)
C6—C1—C7	120.8 (4)	C13—C9—C10	117.2 (4)
C2—C1—C7	120.4 (4)	C13—C9—C8	124.0 (4)
O1—C2—C3	118.6 (4)	C10—C9—C8	118.6 (4)
O1—C2—C1	121.8 (4)	C11—C10—C9	119.6 (4)
C3—C2—C1	119.5 (4)	C11—C10—H10	120.2
C2—C3—C4	121.5 (4)	C9—C10—H10	120.2
C2—C3—Cl1	119.1 (3)	C12—C11—C10	117.3 (4)
C4—C3—Cl1	119.4 (3)	C12—C11—H11	121.4
C3—C4—C5	119.3 (4)	C10—C11—H11	121.4
C3—C4—H4	120.4	N3—C12—C11	125.5 (4)
C5—C4—H4	120.4	N3—C12—Cl3	115.8 (3)
C6—C5—C4	120.6 (4)	C11—C12—Cl3	118.7 (4)
C6—C5—Cl2	120.8 (4)	C12—N3—C13	115.9 (4)
C4—C5—Cl2	118.6 (3)	N3—C13—C9	124.5 (4)
C5—C6—C1	120.3 (4)	N3—C13—H13	117.7
C5—C6—H6	119.9	C9—C13—H13	117.7
C1—C6—H6	119.9		

### Hydrogen-bond geometry (Å, °)

**Table:**

*D*—H···*A*	*D*—H	H···*A*	*D*···*A*	*D*—H···*A*
N2—H2···O2^i^	0.86	2.20	2.930 (4)	142
O1—H1···N1	0.82	1.82	2.540 (4)	147

Symmetry codes: (i) *x*+1, *y*, *z*.
